# Fibrosis

**DOI:** 10.1186/s12967-022-03789-7

**Published:** 2023-01-30

**Authors:** Monica Pernia Marin, Mary Salvatore

**Affiliations:** Columbia University, New York, USA

We are delighted to announce the launch of ***Fibrosis*** a new section of the Journal of Translational Medicine with the purpose of gathering current high-quality research to better understand the process of normal tissue repair as well as the pathogenetic mechanisms responsible for the onset and progression of tissue fibrosis that leads to organ dysfunction and failure. Fibrosis is the common denominator in a variety of chronic diseases including idiopathic pulmonary fibrosis, liver cirrhosis, and ulcerative colitis, among others. These fibrotic diseases affect a vast number of people across the world significantly impacting the quality of their life and increasing health care costs.

Regardless of the organ affected, the dysfunction occurs following the excessive production and deposition of collagen and extracellular matrix by activated myofibroblasts altering the architecture and function of the organ [1]. Oxidative damage, chronic inflammation, and critical signaling cascades have been implicated in the activation of myofibroblasts and the perpetuation of abnormal fibrogenesis [1, 2]. Myofibroblasts are mesenchymal cells with several origins; several experimental studies have described the co-expression of endothelial and mesenchymal cell markers in fibroblasts isolated from animal models of fibrosis suggesting that endothelial cells can differentiate into myofibroblasts during a process called endothelial to mesenchymal transition which is particularly relevant in the pathophysiology of cardiac, renal, and pulmonary fibrosis [3–6].

The pathogenesis of fibrosis is complex and not fully understood, therefore, the development of more effective therapeutic options has been challenging. There are several cell types and signaling pathways responsible for the development of lung fibrosis following repetitive exposure of the alveolar epithelial cells to a variety of injurious stimuli in combination with individual genetic, epigenetic, and immunological characteristics or predisposition [7]. The use of immunosuppressant drugs is controversial [8, 9]. The oral administration of either of the relatively new antifibrotic agents, pirfenidone and nintedanib, has been shown to safely improve quality of life and slow disease progression [10–12].

Liver cirrhosis is an organ-specific fibrosis that compromises important metabolic functions ultimately leading to multisystemic complications and historically death [13]. Liver transplant is still a valuable resource to treat the sickest patients, however, newer antiviral medications have shown promise in restoring liver function and improving life expectancy. These treatment advances in cirrhosis provide encouragement for improved outcomes in other fibrotic diseases.

The goal is early diagnosis and prevention; however, most of these diseases manifest clinically when the affected organ is significantly damaged by fibrotic tissue. The time between initiation of fibrogenesis and symptoms onset varies; during the earliest stages of illness, it is difficult to prognosticate the disease course [14]. The advanced stages of organ-specific fibrosis are usually associated with great deterioration of the overall functional capacity and quality of life with deeply negative implications for the psychosocial health and well-being of patients and caregivers.

Fibrosis has also been further implicated in the proliferation and migration of cancer cells while creating conditions that compromise anti-tumor immunity and treatment response. The collective scientific effort is focused on understanding the interaction of profibrotic molecules and cells with a variety of cancer types aiming to develop anti-fibrotic agents that can also prevent and treat malignancies [15].

The improved understanding of how fibrosis develops, causes morbidity, and promotes cancer is the foundation for making advances in diagnosis, treatment, and ultimately prevention. ***Fibrosis*** will be focused on high-quality research from basic science to clinical trials. The expert members of our Editorial Board are committed to ensuring a productive scientific discussion through the rapid publication of internationally competitive and high-level peer-reviewed articles. We look forward to receiving your thought-provoking contributions to ***Fibrosis.***
